# Preparing African American Men to Make Informed Prostate Cancer Screening Decisions: Development and Pilot Testing of an Interactive Online Decision Aid

**DOI:** 10.2196/15502

**Published:** 2020-05-05

**Authors:** Jennifer Dacey Allen, Amanda Reich, Adolfo G Cuevas, Keren Ladin

**Affiliations:** 1 Department of Community Health Tufts University Medford, MA United States

**Keywords:** decision support techniques, prostate neoplasms, early detection of cancer, decision making (shared), men’s health, minority health

## Abstract

**Background:**

African American men are at a higher risk of developing and dying from prostate cancer compared to white men. The serum prostate-specific antigen (PSA) screening test has a high risk of false-positive results and overdiagnosis; therefore, it is not routinely recommended. Rather, men are encouraged to make individualized decisions with their medical providers, after being fully informed about its potential benefits, limitations, and risks.

**Objective:**

This study aimed to describe the development and pilot testing of an interactive Web-based decision aid (DA; Prostate Cancer Screening Preparation [PCSPrep]) for African American men, designed to promote informed decision making for prostate cancer screening.

**Methods:**

Four focus groups (n=33) were conducted to assess men’s reactions to DAs developed in prior studies and gather information to modify the content and format. The pilot test employed a pre-posttest evaluation design. A convenience sample of 41 men aged 45-70 years with no history of prostate cancer was recruited from community settings. Participants completed online surveys before and after using PCSPrep that assessed prostate cancer screening knowledge, decision self-efficacy, decisional conflict, and preparation for decision making.

**Results:**

Use of PCSPrep was associated with a significant increase in prostate cancer knowledge (49% vs 62% correct responses; *P*<.001), and men also experienced less decisional conflict (24 vs 15 on a scale of 0-100; *P*=.008). No changes in self-efficacy about decision making or screening preferences were observed. Most men (81%) reported that using PCSPrep prepared them to make informed decisions in partnership with their provider.

**Conclusions:**

PCSPrep was an acceptable DA that improved men’s knowledge, reduced decisional conflict, and promoted the perception of being prepared for shared decision making. Further research is needed to test the DA in a larger randomized trial.

## Introduction

### Background

Prostate cancer is the most common (noncutaneous) cancer among men in the United States [1]. Screening with the serum prostate-specific antigen (PSA) test is the main early detection method; it is widely utilized [2] yet remains controversial [3]. At the current time, most medical organizations advise against routine screening because of the potential risks of false-positive test results, overdiagnosis, and overtreatment. Instead, guidelines emphasize the importance of educating men about the potential risks, benefits, and harms of screening and engaging them in a process of shared decision making (SDM) with their providers [4-8]. SDM involves a discussion between the patient and the provider about the best available evidence as well as the consideration of patient preferences and values related to the screening decision [9]. However, national studies show that many patients do not experience SDM in the context of prostate cancer screening decisions [10,11]. SDM is often difficult to accomplish in clinical practice, given the short duration of clinical visits, the need to address competing health priorities, and the communication challenges between patients and providers [12].

For African American men, decision making about prostate cancer screening is particularly complex, as they are 60% more likely to be diagnosed with and nearly 2.5 times more likely to die from prostate cancer compared with white men [13]. African American men are also diagnosed at younger ages and in later stages of disease. No race-based screening guidelines are currently endorsed, and most prostate cancer screening studies have been conducted primarily among white men [14]. However, black race is acknowledged as a significant risk factor for the disease [4]. In light of this, some guidelines recommend that prostate cancer screening be made available to African American men between the ages of 45 to 50 years, but only after they are fully informed about screening [5,7].

Decision aids (DAs) are a promising means to prepare men to engage in SDM and can be administered before a clinical visit. These educational tools provide accurate and unbiased information to inform patients about the potential outcomes of a decision. DAs have generally been found to be effective in increasing patients’ knowledge regarding the disease entity and screening test in question, promoting patients’ sense of self-efficacy with regard to participating in SDM across a wide variety of medical decisions [15]. Over the past decade, there has been a proliferation of DAs for prostate cancer screening [16]. Taken together, these studies demonstrate that DAs are associated with increased knowledge, increased confidence in the ability to engage in decision making and reduced decisional conflict. The majority of these studies were conducted among white men in clinical settings [16].

### Objective

There is a need for DAs for African American men that incorporate disease risk assessment and can be administered in community settings or made available online [17]. This paper describes the development and pilot testing of an interactive, individually tailored Web-based DA designed specifically for African American men (Prostate Cancer Screening Preparation [PCSPrep]).

## Methods

### Conceptual Framework

The Ottawa Decision Support Framework (ODSF) [18], a mid-level theory that guides many decision support studies, provided the framework for the development and evaluation of our DA. The ODSF integrates tenets of multiple theories, including social cognitive theory, social psychology, and decision support to specify modifiable factors that can improve decision making [19]. It addresses an individual’s decision needs (eg, knowledge) and articulates the components of decision support (eg, values clarification) that result in high-quality decisions that are both informed and value-based. Furthermore, the ODSF suggests a step-by-step approach for the decision-making process, which includes identification of available options, acquisition of information and skills necessary for informed decision making and SDM, clarification of values relevant to the decision, and development of a plan for action [18]. See Table 1 for examples of ODSF constructs and how they were addressed in our DA.

**Table 1:** Sample of Ottawa Decision Support Framework constructs, content, and format addressed in Prostate Cancer Screening Preparation decision aid.

Construct	Prostate Cancer Screening Preparation content	Format/presentation
Knowledge	Factual information about prostate cancer incidence and mortality among African American men; potential benefits, risks, and harms of PSA^a^ screening; methods for diagnosing and treating prostate cancer, etc. Users are provided access to a section that assesses individual risk based on the risk factor information input by the user.	Video: Doctors presenting information modeled after a popular television show. Fictionalized audience members “call in” to pose questions, which are subsequently answered by the doctors. Thermometer indicates risk relative to other men of the same age (ie, greater than, similar to, or less than average).
Decision self-efficacy	Users are led through decision-making steps specified by the Ottawa Decision Support Framework, including identifying options, addressing information needs, and clarifying values.	On-screen text with one page per step.
Clarification of values	Consideration of the potential advantages and disadvantages of PSA screening.	Users presented with common “pros” and “cons,” which are rated by users.

^a^PSA: prostate-specific antigen.

### Decision Aid Development Process

The DA development process followed steps and criteria set forth by the International Patient Decision Aid Standards (IPDAS) Collaboration. IPDAS is an international body that offers guidance to enhance the quality and effectiveness of DAs. Guidelines recommend that DAs be (1) developed with feedback from the target audience, (2) provide unbiased and detailed information in lay terms, (3) elicit information about patient needs, and (4) offer structured guidance for deliberation and communication [20]. Steps in our DA development process are described in the sections below.

#### Step 1: Obtain Feedback From the Intended Audience

We conducted 4 focus groups with African American men (n=33) recruited from community settings (eg, churches and barbershops) through fliers and word of mouth (data not shown). Groups were facilitated by a trained African American male moderator. Focus group objectives were to assess men’s reactions to DAs developed in prior studies [21], gather responses to educational messages, and assess preferences for communication strategies (eg, print, video, and online). Participants were men older than 45 years with no prior history of prostate cancer. Focus group audiotapes were professionally transcribed. We performed a thematic analysis including a hybrid of inductive and deductive approaches. First, members of the research team (JA and AR) independently reviewed each transcript and identified initial codes, based on the constructs in the interview guide. Next, team members compared codes, and through an iterative group process, they divided these codes into superordinate and subordinate categories. Following discussion and consensus, team members independently conducted line-by-line coding by compiling themes and descriptive quotes into Microsoft Excel spreadsheets. These documents were reviewed and compared. When there was a disagreement regarding the meaning of a specific quote, members of the team returned to the transcript and/or audiotape to review and come to consensus. Detailed analysis of these data is beyond the scope of this paper. Key themes included a desire for information and graphics specifically targeted for African American men, a preference for African American actors, a need for information, and skills to facilitate engagement in decision making with providers. Physicians and female family members were identified as credible information sources. Of the formats presented (print, video, and online), most men preferred an online tool that included video segments. Delivery on the internet and mobile phones was seen as acceptable among most men.

Working with a team of health communications specialists, Web developers, and experts in prostate cancer screening, we developed storyboards to test 2 potential story lines each of which used an ‘edutainment’ approach [22]. The first was based on a sports show with a known African American sports celebrity. The second was based on a popular network television show in which doctors talk about health issues and invite fictional audience members to ask questions. An additional 2 focus groups were conducted (k=2; n=15) to gather reactions to storyboards. Men’s reactions to the television show concept were more positive than for the sports theme. Many felt that the latter was “not serious enough” and there was no need for a “big celebrity” to highlight the importance of the issue for African American men. Therefore, we proceeded with the talk show story line. The name of the DA, PCSPrep, was deemed acceptable in focus groups.

#### Step 2: Provide Unbiased and Detailed Information in Lay Terms

The content of the DA was based on information covered in our prior DAs [21] as well as a DA developed by the Centers for Disease Control and Prevention titled “Is prostate cancer screening right for you? A decision guide for African Americans” [23], and it included a graphic from the National Cancer Institute [24]. Information was provided through video, on-screen text, and graphics. Topics addressed included the location and function of the prostate gland, incidence of prostate cancer among African American men, risk factors, methods for early detection, potential advantages and disadvantages of screening, the recommendations of major medical organizations that men make individualized decisions, meaning of an elevated PSA test, and methods for diagnosis of prostate cancer. All information was in lay terms, and the on-screen text required only a sixth-grade reading level.

The first section of PCSPrep was a 5-min video in a talk show format hosted by 2 African American doctors (actors). Although men were able to navigate back and forth between DA segments, this video segment could not be bypassed. This was to ensure that men had all the factual information needed for an informed decision. After viewing the video, men were then able to navigate freely between any of the three remaining segments, which are described below.

#### Step 3: Elicit Information About Patient Needs

The second segment of PCSPrep was titled “Learn More.” Here, users could select from a variety of topics to gain more in-depth information (eg, risk of false positive result). The “Learn More” section also included a personalized risk assessment for prostate cancer based on the Your Disease Risk Index [25]. Users input data in response to questions about prostate cancer risk factors (eg, age, family history) and were provided with an on-screen graphic of their risk relative to other men (greater than average, average, less than average) presented as a thermometer, with higher “temperatures” indicating higher risk.

#### Step 4: Provide Structured Guidance for Deliberation and Communication

**Figure 1:**
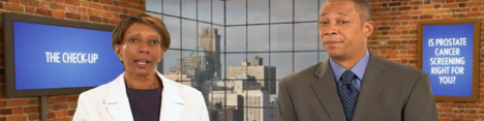
The Check-Up Show.

**Figure 2:**
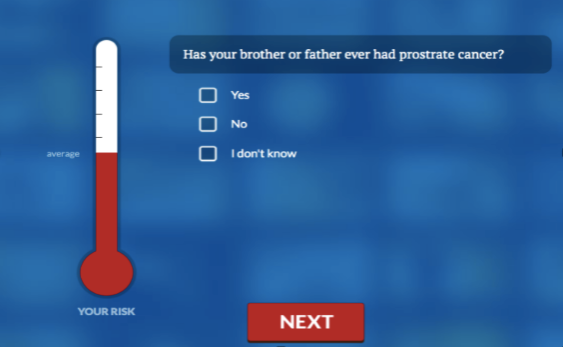
Individualized Risk Assessment.

In the third section (“Decide Now”), men were led through steps of decision making based on the ODSF. Steps included identifying decision options (screen/no screen/decide later), identifying the potential need for additional information (eg, go back to the “Learn More” section or link out to relevant medial websites), and clarifying personal values (ie, “what is most important to me?”). Values clarification included an exercise where men were presented with commonly cited pros and cons of screening (eg, “Screening could give me peace of mind” or “I don’t want to have a PSA test if the results could be wrong”). They were then asked to assign a relative weight to each statement, and their responses were pictorially presented as a scale, with pros on one side and cons on the other. This information was used to guide decision making; if a man had a stated preference to undergo screening but rated the “cons” of screening more heavily than the “pros,” he was told that his decision did not align with his stated values and was encouraged to revisit earlier segments of the DA to get more information and further clarify his values. The fourth segment was titled “Next Steps.” This section included suggestions and tips about how to communicate one’s preference and concerns to a provider as well as information about how to access screening if not otherwise available. It also included a list of questions that one might ask his provider, and a printout of this was given to users. See Figure 1 (depicting television show) and Figure 2 (step in individualized risk assessment).

### Pilot Test of Prostate Cancer Screening Preparation

Men eligible to participate in the pilot test were aged 45 to 70 years, self-identified as African American, had no prior history of prostate cancer, and had not participated in focus groups. We aimed to recruit a total of 50 participants over a 3-month period. Recruitment fliers were placed in a variety of community-based organizations, including churches, barbershops, public housing, and social service agencies. Those interested in participating were screened for eligibility by phone by research assistants.

Eligible men provided written informed consent before data collection and DA use. Men completed online surveys and the DA on a study iPad in a setting that afforded privacy (eg, a meeting room). A research assistant was available to answer questions or provide assistance during survey completion and DA use. Surveys were completed immediately before and following use of the DA. The pretest took approximately 25 min to complete; the posttest took approximately 10 min. The average time spent using PCSPrep was 20 min. Men were provided with a US $50 gift card for their participation. Data collection and intervention administration took place in the summer and fall of 2015. Study procedures were approved by the Institutional Review Boards at the Dana-Farber Cancer Institute and Tufts University.

### Measures

Recognition of the PSA test was assessed with a standard item (“The prostate-specific antigen test (PSA) is a blood test that is used to find prostate cancer. Before now, had you ever heard of the PSA test?”). Subsequently, men were asked about their history of PSA testing (“Have you ever had a PSA test?” and if so, “When did you have your last PSA test?”). Assessments of the primary informed decision-making outcomes are described below. All scales with multiple survey items assessing latent constructs had good internal reliability in this sample (Cronbach alphas ranging from 0.70 to 0.91; see Table 2).

Prostate cancer knowledge was assessed with 14 questions from a validated prostate cancer knowledge scale [26]. Questions included the incidence of prostate cancer, risk factors, screening modalities as well as their limitations (false positives), diagnostic procedures, and potential treatment-related complications. The proportion of correct responses was divided by 14 to create a 0-100% scale, with higher scores indicating greater knowledge.

Decision self-efficacy*,* or confidence in the ability to make an informed decision and to participate in the decision making at a personally desired level, was assessed with the 11-item Decision Self-Efficacy Scale [27]. Questions ask the respondent to reflect about how confident they feel about various aspects of the decision-making process (eg, “I feel confident that I can get the facts that I need to make an informed choice”), with 3 response options including “very confident,” “somewhat confident,” and “not at all confident.” Scores are summed, divided by the total number of items, and multiplied by 25, to arrive at a range of scores from 0 (low self-efficacy) to 100 (high self-efficacy).

**Table 2:** Characteristics of study participants in the Prostate Cancer Screening Preparation pilot study (n=41).

Characteristics		Value, n (%)^a^
**Age (years)**		
	45-54	16 (43)
	55-64	15 (41)
	65-70	6 (16)
**Household income (US $)**		
	Less than $25,000	12 (29)
	$25,000-$49,999	8 (20)
	$50,000-$74,999	13 (31)
	More than $75,000	8 (19)
**Marital status**		
	Not married	21 (51)
	Married/living as married	20 (49)
**Educational level**		
	Less than high school	2 (5)
	Some college or 2-year degree	10 (23)
	4-year college degree	17 (41)
	More than a 4-year college degree	12 (29)
**Prior prostate-specific antigen screening**		
	Yes	35(85)
**Computer skills**		
	Very good/good	24 (59)
	Fair/poor	17 (41)

^a^Total varies because of missing responses; percentages may not total to 100% because of rounding.

Value of screening was assessed with 8-items from our prior studies [21,28]. Participants were asked to rate their extent of agreement with statements about potential advantages (eg, “I will have peace of mind if I have prostate cancer screening”) and disadvantages of screening (eg, “I do not want to have a PSA test unless doctors are reasonably certain that it can save lives”) on a 4-point Likert scale (strongly agree to strongly disagree), with higher values indicating stronger agreement. Before standardizing the values scale (0-100), negative items were reverse coded such that high scores indicate a greater value placed on screening.

The Decisional Conflict Scale includes items that assess the degree to which an individual feels informed to make a decision consistent with his values, experiences uncertainty in choosing options (eg, feeling uninformed and unclear about personal values), and is likely to implement the decision. We used the low-literacy version of the scale, with 10 items, which has demonstrated good reliability and validity [29]. Questions include “Are you clear about the best choice for you?” and “Are you clear about which benefits matter most to you?” (yes/no/I don’t know). Scoring is such that 0 represents no conflict and 100 reflects the highest level of conflict.

Preparedness for decision making, asked only at posttest, included a validated scale with 8 items assessing the degree to which the DA helped to provide information to make an informed decision [30]. For example, men were asked the extent to which PCSPrep helped them to *recognize* that a decision needed to be made about screening and whether they felt prepared to talk with their provider about their values related to screening. Response options were rated on a 5-point scale from “a great deal” to “not at all,” with standardized higher scores (0-100) indicating greater preparedness.

We also assessed the perceived risk of prostate cancer because the DA highlighted elevated risk among African American men. This was assessed with 2 items from prior studies [31]. The first inquired about overall risk: “How likely do you think it is that you will develop prostate cancer in the next 5 years?” (very likely to very unlikely). The second asked, “Compared to the average man your age, would you say that you are...?,” with response options including “more,” “less,” and “the same”.

Decisional status was assessed by asking “If you had to decide now, what would you choose?,” with response options including “I have decided to be/not to be screened” and “I don’t know.” Demographic characteristics, screening history, and access to health care were also assessed, using standard items from the Behavioral Risk Factor Surveillance Surveys [32].

### Analysis

Descriptive statistics were calculated to assess participants’ sociodemographic characteristics and prostate cancer screening behaviors. Paired *t* tests were performed to assess changes in continuous variables (eg, knowledge and decision self-efficacy) between pretest and posttest. The McNemar test was used to assess changes in decisional status (decided or undecided) and screening preference. The Wilcoxon signed-rank test was used to assess changes in perceived risk between pretest and posttest. Cronbach alpha was used to assess the internal reliability of multi-item scales that assessed latent constructs. Effect sizes were calculated as observed average difference (delta=post minus pretest scores) divided by the standard deviation of the difference.

## Results

### Characteristics of the Study Sample

A total of 41 eligible men were recruited to the study over a 3-month period, 82% of our recruitment goal. Participants were aged between 45 and 70 years. About half the participants were married or living as married and had annual household incomes of less than US $50,000. More than two-thirds of the participants had completed a college degree or higher. A majority of the participants had heard of the PSA test and reported that they had undergone PSA screening in the past (85%). Most (78%) of the participants who had undergone PSA testing reported that they knew the results of testing, with only 7% (n=1) reporting that their PSA level had been above the normal range. About two-thirds (61%) of the participants had heard of the digital rectal exam, and 74% of the participants had undergone one in the past. Over half of the participants reported that their computer skills were “good” or “very good,” and none of the participants required assistance with use of PCSPrep from the research assistant. See Table 2.

### Changes From Pretest to Posttest

Effect sizes for changes in knowledge, decisional conflict, and perceptions about the value of screening were moderate. Results are presented in Table 3. Specifically, knowledge about prostate cancer and available screening methods was very low at pretest and improved significantly after the use of PCSPrep (49% to 62%, *P*=.001; effect size=0.56). Confidence in the ability to make an informed decision (self-efficacy) was high at baseline and did not change after use of the DA (86 vs 88; *P*=.84). After using PCSPrep, men reported having lower levels of decisional conflict about screening (24 vs 15 on a scale of 0-100; *P*=.008; effect size=−0.44). Men’s perceptions about the advantages of screening were high but decreased after the use of the DA (74 to 71 on a scale of 0-100; *P*=.02; effect size=−0.38). At posttest, fewer men rated their risk of developing prostate cancer to be lower than men of the same age, but this was only marginally significant (75% to 67%; *P*=.08).There was no change in the percentage of men who believed that they were “certain” or “very likely” to develop the disease in the next 5 years (data not shown). Most men (81%) reported that using PCSPrep prepared them “very well” or “well” to make informed decisions in partnership with their provided (data not shown).

**Table 3:** Changes in informed decision-making outcomes from pretest to posttest in the Prostate Cancer Screening Preparation pilot study (n=41).

Informed decision-making outcomes	Internal reliability Cronbach alpha	Pretest, mean (SD)	Posttest, mean (SD)	Change, *P* value	Effect size	
Knowledge (0-100)	.77	49.45 (21.52)	61.94 (19.97)	*.001* ^a^	0.56	
Decision self-efficacy (0-100)	.90	86.12 (18.60)	88.51 (16.89)	.84	N/A^b^	
Decisional Conflict Scale (*r* 0-100); Cronbach alpha=.86	.86	23.8 (26.6)	14.8 (19.52)	*.008* ^a^	−0.44	
Value of screening (0-100)	.75	75.7 (13.96)	70.67 (15.73)	*.01* ^a^	−0.38	

^a^*P*<.05.

^b^N/A: not applicable.

### Screening Decision and Preferences

There were no changes in men’s screening preferences before and after using PCSPrep; at pretest, 46% of men said that they had made a definitive decision, and 47% of men reported this to be the case at posttest. The vast majority of men preferred to be screened (86%), and this did not change between test pre-test and posttest (data not shown).

## Discussion

### Principal Findings

DAs have been found to be effective interventions to complement patient/provider engagement in SDM by providing patients with information needed to assess their options and examine their values as they relate to those options. Indeed, the Centers for Medicare and Medicaid Services now requires SDM for some preference-sensitive conditions [33]. One way to achieve this is through the use of DAs. However, the development of DAs and use among African American men is incompletely understood. For African American men, the decision to undergo or forgo screening poses challenges, given their elevated risk for the disease and the controversial nature of the PSA test. It has been suggested that offering DAs for prostate cancer screening outside of a clinical setting may be particularly important for African American men who report difficulty communicating with medical providers and may have a high level of medical mistrust [34].

To our knowledge, this is the first interactive, online DA developed specifically for African American men that provides individualized risk assessment. We found PCSPrep to be feasible to administer in community settings, even among those who reported low levels of computer skills. Moreover, men reported high levels of agreement when asked the extent to which PCSPrep helped prepare them to organize their thinking, make decisions, and have conversations about screening with their providers (ie, “preparedness for decision making”).

After using PCSPrep, men had significantly greater knowledge about prostate cancer screening. However, improvements in knowledge did not translate into changes in screening preferences. Our finding of increased knowledge is consistent with other DA interventions among general audiences [35,36] and among African American men [28,37,38]. Our finding of an average 13 percentage point increase in prostate cancer knowledge is in line with these previous reports [28]. Nevertheless, having the knowledge deemed necessary to make an informed decision was still suboptimal after using the tool. Prior studies of prostate cancer screening knowledge among African American men have similarly found low levels of the knowledge required for informed decision making [28,37].

We also found that the DA did not change men’s self-efficacy about making informed decisions. Men had very high levels of confidence in their ability to make informed decisions before using the DA, despite having relatively low levels of knowledge. Knowledge scores were not correlated with decision self-efficacy (Pearson *r*=−0.25; *P*=.12). We believe that further attention to the relationship between knowledge and decision self-efficacy is warranted in future studies.

After engaging with PCSPrep, men perceived fewer advantages of screening, a phenomenon that has been consistently reported [16]. Despite this, overall opinions about screening were universally favorable, with the majority valuing the benefits over the potential risks and harms. Similarly, other studies have also found that men tend to prefer prostate cancer screening even in light of its limitations. Indeed, few people are aware of the concept of over detection or can identify potential harms of screening [39,40], and few people decline screening even when provided with information about risks of false-positive test results [41,42].

We found that men had less decisional conflict after using PCSPrep, which is also consistent with prior DA studies [35,36]. Presumably, improvements in knowledge and clarification of preferences reduces men’s ambivalence about decision making and improves decision quality. A study using structural equation modeling found that increased knowledge after DA use had an indirect effect on decisional conflict by increasing the perceived risk and decreasing anxiety about decision making [43]. In our study, men gained significant knowledge and were more likely to perceive themselves to be at a higher risk compared with men of the same age. At the same time, decisional conflict was reduced. However, we did not find knowledge to be significantly correlated with decisional conflict at posttest (Pearson *r*=−0.24; *P*=.13). Future research should examine other intraindividual factors, such as cultural barriers and decision-making preferences, that may play a role in decisional conflict.

### Strengths and Limitations

This study has limitations that must be acknowledged. First, we used a quasi-experimental design with a small convenience sample with no control group. We cannot rule out alternative explanations for findings nor can we generalize these results to the broader population of African American men. Moreover, we did not assess actual screening behaviors, men’s actual discussions with their providers, or long-term retention of knowledge or skills. These are limitations in the existing literature, and these longer-term outcomes warrant further study. We also acknowledge that men in this sample were more highly educated than the general US population [44], with two-thirds of the men having completed at least a bachelor’s degree. Although we designed the DA for a population with a sixth-grade reading level, we cannot assume that it would have the same impact in a sample with lower levels of education.

Despite these limitations, we believe that this study can offer some new insights for prostate cancer screening DAs. This is among the first DAs designed for African American men that integrate personalized risk estimates and other interactive functions, including values clarification exercises. We also specifically addressed the issue of false-positive test results in the DA, which has been called for in two recent systematic reviews of prostate screening DAs [35,36]. These features may enhance attention to messages, increase understanding and recall, and potentially lead to improved quality of decisions. If the DA is ultimately found efficacious in a larger randomized controlled trial, the online format has the potential to reach broad, geographically dispersed populations.

Moreover, this paper is responsive to the recent calls for the explicit articulation of theoretical underpinnings for DA interventions [45]. The application and testing of theoretical models could enhance the understanding of the mechanisms through which DAs operate and ultimately improve their efficacy. We observed that fewer men believed that their risk of developing prostate cancer compared with men in the same age group increased but their perceived risk of developing the disease in the next 5 years was unchanged. This suggests that decision support may improve the accuracy of disease risk among this population, and at the same time, enable men to make decisions without undue internal conflict. Future research is needed to determine if existing conceptual models for decision support interventions, which tend to emphasize knowledge acquisition, can have a greater impact on other decision-making outcomes, including actual screening decisions and engagement in SDM with providers.

## Abbreviations

DA: decision aid
IPDAS: International Patient Decision Aid Standards
ODSF: Ottawa Decision Support Framework
PCSPrep: Prostate Cancer Screening Preparation
PSA: prostate-specific antigen
SDM: shared decision making
